# Healthcare Providers’ Perceptions of Challenges with Frequent Users of Emergency Department Care in Switzerland: A Qualitative Study

**DOI:** 10.1177/00469580211028173

**Published:** 2021-07-30

**Authors:** Patrick Bodenmann, Miriam Kasztura, Madison Graells, Elodie Schmutz, Oriane Chastonay, Marina Canepa-Allen, Joanna Moullin, Michael von Allmen, Melissa Lemoine, Olivier Hugli, Jean-Bernard Daeppen, Véronique S. Grazioli

**Affiliations:** 1 Department of Vulnerabilities and Social Medicine, Center for Primary Care and Public Health, Chair of Medicine for Vulnerable Populations, University of Lausanne, Switzerland; 2 Faculty Health Sciences, School of Pharmacy and Biomedical Sciences, Curtin University, Western Australia; 3 Emergency Department, Lausanne University Hospital, University of Lausanne, Switzerland; 4 Addiction Medicine, Department of Psychiatry, University of Lausanne, Switzerland

**Keywords:** frequent users of emergency department, ED healthcare providers’ challenges

## Abstract

Frequent users of emergency departments (FUED; ≥ 5 ED visits/year) commonly cumulate medical, social, and substance use problems requiring complex and sustained care coordination often unavailable in ED. This study aimed to explore ED healthcare providers’ challenges related to FUED care to gain insight into the support and resources required to address FUED complex needs. An online survey was sent to all general adult emergency services within Switzerland (N = 106). Participants were asked to indicate the extent to which they perceived that FUED represented a problem and to describe the main challenges encountered. In total, 208 physicians and nurses from 75 EDs (70.7%) completed the survey. Among the 208 participants, 134 (64%) reported that FUED represented a challenge and 133 described 1 to 5 challenges encountered. A conventional content analysis yielded 4 main categories of perceived challenges. Negative consequences in the ED secondary to FUED’s presence (eg, ED overcrowding, staff helplessness, and fatigue) was the most frequently reported challenge, followed by challenges related to FUEDs’ characteristics (eg, mental health and social problems) leading to healthcare complexity. The third most frequently encountered challenge was related to the ED inappropriateness and inefficiency to address FUEDs’ needs. Finally, challenges related to the lack of FUED healthcare network were the least often mentioned. ED healthcare providers experience a wide range of challenges related to FUED care. These findings suggest that currently EDs nor their staff are equipped to address FUEDs’ complex needs.

**What do we already know about this topic?**
Frequent users of emergency departments (FUED; ≥ 5 ED visits/year) commonly cumulate medical, social, and substance use problems requiring complex and sustained care coordination often unavailable in ED and typically receive ED care that is less satisfying to them than infrequent ED users.
**How does this research contribute to the field?**
Findings indicate that ED healthcare providers experience a wide range of challenges related to FUED cares. Findings provide a broader understanding of challenges related to FUED and the needs and supports required for ED staff to address FUED complex needs.
**What are research’s implications toward theory, practice, or policy?**
Providing ED staff with support and tailored tools to help them address FUED complex needs may be accomplished by implementing case management intervention tailored to FUED in ED.

Frequent users of emergency departments (FUED; ≥ 5 ED visits/year) have been a focus of attention in emergency medicine for more than 3 decades in developed countries.^1,2^ Although they represent a small number of patients, they account for a disproportionately high number of all ED visits^3^ and are therefore often considered contributors to ED overcrowding.^1^ In Switzerland, a study conducted at the Lausanne University Hospital showed that FUED represented 4.4% of all ED patients, accounting for 12.1% of all ED visits.^3^ Like most developed countries, Switzerland has universal health coverage, relying on mandatory individual health insurance, enabling access to care.

Driving the high number of ED visits is the fact that FUED often cumulate chronic medical diseases, psychological, substance use, and social problems.^3-6^ Despite their need of services, FUED receive ED care that is less satisfying to them than infrequent ED users.^7^ Past qualitative research conducted in Sweden and in Canada provide insight into their common dissatisfaction, revealing that FUED perceived ED staff to consider their demands as time-consuming and inappropriate whereas, they viewed them as urgent.^8-10^ Hence, they perceived ED staff to belittle their symptoms and did not feel understood.

In response, previous research focused on identifying, developing, and testing interventions tailored to FUED highlighted case management (CM) as one of the most promising approaches.^2^ Consistent with FUED needs, CM is a collaborative intervention that aims to ensure and coordinate tailored care and services on the basis of a holistic evaluation of patients’ needs and priorities.^11,12^ A growing body of research supports CM effectiveness in reducing ED use and related costs while improving housing and environmental quality of life among FUED.^11,13,14^

Despite these promising findings, wide-spread implementation across different care settings, such as community hospitals and non-academic centers, remains uncommon. To help address this gap between the evidence of CM effectiveness and its use in practice, research providing insight into potential strategies to implement CM is needed.^2^ To contribute to this research agenda, our team is currently conducting a project aimed at implementing CM tailored to FUED in public hospitals within the ED in the French speaking part of Switzerland.^15^ The first step of this ongoing study was to explore ED staffs’ perceptions of FUED and gauge their interests in implementing CM. An online survey was sent to all general adult emergency services in Switzerland.^16^ In total, 64% of participants perceived important challenges around FUED. Findings also indicated that the majority of ED staff face FUED regularly, yet they feel poorly informed on how to manage them. Interestingly, whereas more than 90% of ED staff thought that CM could be useful, less than 60% showed interest in implementing it.^16^ Hence, despite a perceived need and interest in implementing CM, ED staff appear to face barriers adopting and implementing it.

These findings suggest that in order to scale-up CM across Switzerland, developing strategies to increase ED staff willingness to implement CM are necessary. Improved knowledge of ED staff experience related to FUED may assist in developing such strategies. That said, surprisingly, scarce attention has been paid to ED healthcare providers’ experience related to FUED. A recent qualitative study conducted in Sweden explored the experience of encounters with frequent users of psychiatric ED among nurses and physicians.^17^ The main findings revealed that psychiatric ED nurses and physicians found their encounters with frequent users as “caring, professional, and human processes” requiring specific abilities, such as self-awareness, self-acceptance, and self-compassion. Another qualitative study conducted with the same sample explored ED staff understanding of frequent users and their needs.^18^ Nurses and physicians perceived that psychiatric FUED frequent users needed to be relieved from loneliness, hopelessness, and psychiatric symptoms with cohesive care and support.

Whereas these findings provide interesting insight into the experience of ED healthcare staff with FUED, to the best of the authors’ knowledge, there are only 2 qualitative studies exploring their perceived challenges related to FUED care. The first study involved ED nurses in the USA.^19^ Echoing findings in FUED described above, findings revealed that ED nurses were concerned with the appropriateness of FUEDs’ reasons to visit ED. Findings also documented a perception of mismatch between FUED demands and the ED biomedical orientation, leading to shared feelings by both ED staff and FUED of failure, reduced moral and frustration. The second study was conducted in Singapore and involved ED staff perceptions of frequent users of psychiatric ED.^20^ Mirroring parts of the findings described above, results revealed challenges in addressing patients’ needs without suitable alternatives, leading to feelings of failure and fatigue.

These findings provide an initial indication that ED staff encounter challenges related to FUED care, suggesting that FUED phenomena represents a larger problem affecting both FUED and ED clinicians. Gaining a better knowledge of the specific challenges faced by both ED physicians and nurses is important to identify which supports and resources are needed to address both the complex needs of FUED and develop strategies to implement them into practice. In response, this study aimed to explore nurses and physicians’ perceptions of challenges related to FUED care in ED public hospitals in Switzerland.

## Materials and Methods

### Procedures

The data were collected as part of the larger ongoing research project aiming at implementing a case-management intervention tailored to FUED in public hospitals with ED in the French speaking part of Switzerland.^15^ As mentioned earlier, the first step of this larger study was to explore perceptions of FUED and gauge interests in implementing CM by ED staff. To do so, an online survey was sent across all general adult emergency services in the French part of Switzerland. To evaluate broader interest in implementing CM, the survey was further sent across the whole of Switzerland (106 hospital sites). The survey was sent with a short explicative text about FUED and CM to heads of ED of the 106 hospitals. Recipients were asked to forward the survey to their ED colleagues (ie, head nurses, nurses, chief residents, and residents). The assessment took place between September 2017 and March 2018. The survey was anonymous; accordingly, participants did not provide informed consent. Participants were informed that the survey was part of the larger Swiss National Science Foundation (FNS 407440_167341) funded research project and that data would enable a national picture of FUED problem. All procedures were approved by the Human Research Ethics Committee of Canton de Vaud (2018-00442).

### Measures

The larger survey included 19 closed and open-ended questions, in German, French, and Italian, (covering 99.2% of the Swiss population) assessing ED staff’s perception of FUED and interest in implementing CM.^16^ Of those, the 2 questions exploring ED staff perceptions of challenges related to FUED care were used in the current study. Participants were asked to indicate the extent to which they perceive FUED to be problematic within their ED service, using a Likert scale ranging from 1 = *a non-important problem* to 4 = *an extremely important problem*. Next, an open-ended question asked participants to describe the main challenges encountered (ie, *If yes* [if you answered yes to the previous question], *what are the main challenges you encounter?*).

### Participants

Of the 106 sites invited to participate, 75 sites (70.7%) completed and sent back at least 1 survey (range of responses by site: 1-19).The initial sample included 248 respondents. Missing data (n = 39; 15.7%) on key variables for the current study were listwise, resulting in a finale sample of 209 participants.

### Qualitative Analysis

This study entails a qualitative description. The aim of qualitative description is to provide a comprehensive summary of the qualitative data, while staying close to the words. This method was selected because it enables to obtain straight and non-theorized answers to questions relevant for practitioners, researchers, or policy makers.^21^ Accordingly, conventional content analysis was used to analyze responses to the open-ended question exploring challenges related to FUED care.^22^ This methodology facilitates description and summary of qualitative data through a systematic process of coding and classification. Specifically, participants’ responses were reviewed by a master’s-level nurse and a PhD-level psychologist to identify recurring categories of challenges.^22^ Initial coding was conducted independently and a codebook was created in consensus meetings, pooling codes, and eliminating idiosyncratic or redundant codes. The codebook was reviewed by a master’s-level clinician nurse and adapted in consensus meetings, resulting in the final version. Next, 2 raters (post-baccalaureate and PhD-level researchers) independently rated all responses. Interrater reliability was established using percent agreement (82%) and reached acceptable levels according to standards in the literature.^23^ Remaining discrepancies were resolved in consensus meetings.

Frequency analysis was conducted in SPSS 26 to describe the endorsement of different challenge categories in the complete sample and by professions (ie, physicians, nurses). This final step aimed to provide insight into which challenges are most frequent and whether challenges differ between professions and management positions.

### Reflexivity

We conducted the analysis recognizing that participants and researchers typically construct their own perceptions through their previous experiences.^24^ Members of the research teams have expertise and/or experience with FUED. Instead of denying those, they were considered during the analysis process. Specifically, members of the research team involved in the analysis were asked to reflect on their own perceptions and consider them while proceeding the initial coding and developing the final codebook.

## Results

### Description of the Sample

More than half of the initial sample (52.6%) came from the German speaking part of Switzerland, whereas 43.6% and 3.8% were from the French and Italian speaking parts, respectively. The number of respondents fit approximately with the proportion of residents in each part of Switzerland (ie, 71%, 24.5%, and 4.5% in the German, French, and Italian parts, respectively) and contacted hospitals (ie, 72%, 21%, and 7% in the German, French, and Italian parts, respectively), even though the French part was slightly overrepresented. Although there are some cultural differences across linguistic regions in Switzerland, the overall healthcare system is similar nationwide. Specifically, all linguistic regions in Switzerland have universal health coverage. This system relies on mandatory individual health insurance with government subsidies where necessary. The complete sample included 48 nurses (23.1%), 45 head nurses (21.6%), 96 head physicians (46.2%), and 19 residents (9.1%).

Of the 208 participants, 133 provided at least 1 answer to the open-ended question asking participants to describe the main challenges encountered (458 answers in total). Among the 75 participants who provided no answer to the open-ended question, 62 participants (82.7%) perceived FUED to be a non-important problem, 11 (14.7%) a rather-important problem and 2 (2.7%) an important problem. Table 1 displays descriptive statistics of the subsample of participants who provided at least 1 answer to the open-ended question (n = 133).

**Table 1.** Descriptive Statistics for the Subsample of Participants Providing at Least 1 Qualitative Answer (*n* = 133).

Variable	*n* (%)
Profession	
Nurse	32 (24%)
Head nurse	30 (22.6%)
Physician	13 (9.8%)
Head physician	58 (43.6%)
Management position	
Management position	88 (66.2%)
No management position	45 (33.8%)
Perception of FUED problem	
Non-important problem	13 (9.8%)
Rather-important problem	74 (55.6%)
Important problem	41 (30.8%)
Extremely important problem	5 (3.8%)

### Qualitative Results

Content analysis yielded 4 main categories of perceived challenges related to FUED: “negative consequences related to the presence of FUED in the ED” was the most encountered category (39.7%), followed by “challenges related to FUED characteristics” (26.9%), “ED inappropriateness and inefficiency to address FUED issues” (24.5%) and “challenges related to the lack of FUED healthcare network” (9%). As shown in Table 2, the rank order of category frequency was consistent between professions (ie, physicians and nurses) and for ED staff with management positions. However, among ED staff without management positions, the second and third most encountered categories were “ED inappropriateness and inefficiency to address FUED issues” and “challenges related to FUED characteristics,” respectively. Furthermore, results indicated some differences in the rank order of challenge frequency within each category between professions and between management positions. Accordingly, we describe below each category of challenge in 2 steps: (a) the difficulties encountered within the complete sample and (b) the main differences in the rank order of the top 3 challenge frequencies between professions and management positions. Table 2 displays the frequencies of responses within category by professions management positions and provides additional examples not shown in text.

**Table 2.** Perceived Challenges Related to FUED in Rank Order of Frequency by Management Position and Professions.

Category	Examples	All *N* = 133			Management *n* = 88			Non-management *n* = 45			Nurses *n* = 62			Physicians *n* = 71		
		*F* ^1^	%	*R* ^2^	*F* ^1^	%	*R* ^2^	*F* ^1^	%	*R* ^2^	*F* ^1^	%	*R* ^2^	*F* ^1^	%	*R* ^2^
Negative consequences in the ED		182	39.7	1	121	40.3	1	61	38.6	1	91	43.5	1	91	36.6	1
ED resources used by FUED	“Significant investment of time,” “costs,” “use of resources,” “requires a lot of staff”	62	34.1		50	41.3		12	19.7		19	20.9		43	47.3	
ED staff helplessness and fatigue	“Feelings of helplessness”	28	15.4		17	14		11	18		18	19.8		10	11	
Risk of trivializing	“Fear of missing a serious problem,” “objectivity bias”	22	12.1		16	13.2		6	9.4		15	16.5		7	7.7	
Consequences on ED functioning	“ER bed occupancy for many hours”	22	12.1		14	11.6		8	13.1		10	11		12	13.2	
ED overcrowding	“Service overload”	18	9		7	5.8		11	18		13	14.3		5	5.5	
Consequences for other ED patients	“Increase in waiting time”	15	8.2		10	8.3		5	8.2		6	6.6		9	9.9	
Work overload	“Non-urgent administrative work overload,” “it overloads healthcare providers”	9	4.9		3	2.5		6	9.4		8	8.8		1	1.1	
FUED-staff relationship challenges	“Risk of negative attitude [toward FUED]”	6	3.3		4	3.3		2	3.3		2	2.2		4	4.4	
Challenges related to FUED characteristics		123	26.9	2	81	27	2	42	26.6	3	53	25.4	2	70	28.1	2
FUED behavior	“Demanding behavior,” “sometimes impatient et aggressive”	40	32.5		24	29.6		16	38.1		29	54.7		11	15.7	
FUED health condition complexity	“Multi-morbid elderly patients,” “difficult to identify the current diagnostic”	17	13.8		15	18.5		2	4.8		4	7.5		13	18.6	
FUED mental health problems	“Psychiatric hospitalizations”	15	12.2		7	8.6		8	19		4	7.5		11	15.7	
FUED recurrent ED use	“Repeated hospitalizations,” “repetitive huge efforts”	14	11.4		10	12.3		4	9.5		5	9.4		9	12.9	
FUED chronic health problems	“Chronic obstructive pulmonary disease,” “recurrent problems”	13	10.6		8	9.9		5	11.9		4	7.5		9	12.9	
FUED social problems	“Social hospitalizations”	9	7.3		6	7.4		3	7.1		3	5.7		6	8.6	
FUED substance use problems	“Some of them with chronic consumption of substances”	5	4.1		4	4.9		1	2.4		2	3.8		3	4.3	
FUED culture and health literacy	“Comprehension problems in oral”	5	4.1		3	3.7		2	4.8		2	3.8		3	4.3	
FUED ED visit timing	“Visits during off-peak hours”	5	4.1		4	4.9		1	2.4		-			5	7.1	
ED inappropriateness and inefficiency to address FUED issues		112	24.5	3	68	22.7	3	44	27.8	2	49	23.4	3	63	25.3	3
ED healthcare inefficiency	“Challenges to provide adequate care,” “Failure to resolve the patient’s initial problem”	41	36.6		29	42.6		12	27.3		15	30.6		26	41.3	
ED not tailored to FUED needs	“ED is not the right place for these patients,” “they often visit with trifles”	28	25		13	19.1		15	34.1		16	32.7		12	19	
ED over-investigation	“Repetitive investigations,” “exaggerated consumption of clinical exams”	18	16.1		9	13.2		9	20.5		7	14.3		11	17.5	
ED healthcare futility	“Resources allocated to unnecessary things,” “often unnecessary occupation of ED”	16	12.3		12	17.6		4	9.1		6	12.2		10	15.9	
Lack of FUED healthcare procedure	“Lack of guidelines”	6	5.4		5	7.4		1	2.3		4	8.2		4	6.3	
FUED dissatisfaction	“Dissatisfaction with care”	3	2.7		-			3	6.8		1	2		2	3.2	
Lack of FUED healthcare network		41	9.0	4	30	10	4	11	7	4	16	7.7	4	25	10	4
Challenges related to FUED healthcare network	“Follow-ups of chronic conditions without GP,” “lack of longitudinal healthcare structure,” “we fulfill the lack of means outside the ED”	41	100		30	100		11	100		14	100		25	100	
Total		458			300			158			209			249		

*Note.* Percentages may not total 100% due to rounding.

### Negative Consequences Related to the Presence of FUED in the ED

The most frequently cited negative consequence was related to the resources used by FUED. Participants also commonly pointed to the time spent on FUED healthcare, noting for instance that they [FUED] require “an important investment in time.” Relatedly, some participants mentioned that FUED demand staff availability (eg, “requires healthcare by experienced physicians”). Participants also frequently emphasized the costs of FUED healthcare, leading to “increases in public health costs.”

Staff helplessness and fatigue represented the second most common perceived negative consequence related to FUED. Some participants reported “staff fatigue” in general, whereas others mentioned feelings of “demotivation,” “weariness,” and “discouragement” among ED healthcare providers. Relatedly, a feeling of helplessness by staff was frequently cited (eg, “feeling of failure for healthcare providers”).

The third most frequently endorsed negative consequence was the risk of trivializing healthcare issues. Participants commonly mentioned the risk of losing objectivity in healthcare (eg, “the patient is no longer taken seriously”). Relatedly, many participants expressed fears of missing a serious medical problem (eg, “missing a proven diagnosis, for once”) or underestimating the seriousness of the situation (“situation underestimated regarding its severity”).

The 2 next most frequently cited negative consequences included ED overcrowding and consequences on ED functioning. ED overcrowding included perceived difficulties such as “increased flow” or “flow overload.” Consequently, some participants evoked organizational consequences in the ED related to the presence of FUED (eg, “disrupts ED functioning”), commonly related to “time management” issues (eg, “box care occupancy for long hours”). Similarly, the next subcategory included negative consequences for other patients. Some participants mentioned that the presence of FUED lengthens “waiting time” or “ED stay” for other patients. More generally, FUED were perceived as using ED resources to the detriment of other “true emergencies” (eg, “They [FUED] block ED service for healthcare management of more urgent cases”).

The 2 least cited perceived negative consequences were work overload and difficulties in building a relationship with FUED. Participants sometimes noted work overload related to the presence of FUED (eg, “overload of non-urgent administrative work”). Finally, a few participants mentioned negative consequences on the relationship between FUED and healthcare providers, evoking risks of endorsing “negative attitudes toward these patients” and ultimately “decreased” or “loss of empathy.”

#### Frequency rank-order differences between professions

Among the top 3 negative consequences related to FUED in the ED, resources used by FUED and ED staff helplessness and fatigue stood in the 2 first positions for nurses, physicians, and ED staff with and without management positions. Next, was the risk of trivializing healthcare issues for nurses and ED staff with management positions, whereas it included negative consequences on ED functioning among physicians and ED overcrowding in ED staff without management positions.

### Challenges Related to FUED Characteristics

Participants reported FUED characteristics leading to healthcare complexity as a challenge. Among the reported characteristics, perceived challenges related to FUED behavior represented almost half of the responses. A demanding attitude was frequently cited among participants, who expressed for instance “lots of expectations,” or “higher demands in healthcare” among FUED. Close to this idea, “impatience” among FUED was another common answer. Less frequent answers included “aggressiveness” or “inadequate and unreasonable behavior.”

Perceived challenges related to the complexity of FUED health condition(s) made up the next most frequently cited answers within this category (eg, “complex psychiatric patients,” “complex and non-curable diseases”). Answers within this subcategory also included complexity related to FUED co-morbidities (eg, “multi-morbid patients with many drugs and interactions”) and to FUED diagnostic identification (eg, “hypothetical multiple diagnoses”). Healthcare complexity was also related to FUED mental health problems, which made up the next subcategory. Participants frequently reported mental health problems of FUED as a challenge (eg, “very anxious patients”) and the lack of means to treat them (eg, “psychiatric patient visiting recurrently the [ER] for a somatic motif without any possibility to orient him to psychiatric cares”).

Perceived challenges related to FUED recurrent ED use was the fourth most common answer within this category. Answers within this subcategory reflected challenges related to the repetition itself (eg, “the problem of the *déjà vu*,” “same care for a recurrent demand”) or to the repetition of arduous situations (“helplessness facing recurrent demand”).

Perceived challenges related to the medical and social problems among FUED represented the next most frequently endorsed subcategories. Participants evoked medical and social problems leading to healthcare complexity and challenges. Among those, social issues (eg, “social misery without any possibility to plan a social assistance in the ER”) and chronic health problems (eg, “back pain”) were the most often reported, followed by substance use related problems (eg, “drug request,” “patient with alcohol or drug problems: recurrent visits with aggressiveness”).

The next most frequently reported subcategory referred to culture and health literacy. This subcategory included challenges related to communication and understanding issues among FUED. Participants pointed to the fact that some FUED speak a foreign language, leading to “communication difficulties” with healthcare providers or “listening problems.” Other participants noted low health literacy among FUED, manifesting itself in a lack of understanding of the healthcare system (eg, “repeated visits in the ER despite in-depth explanations,” “lost patients”) or encountered health problems (eg, “they [FUED] cannot appraise their own health condition objectively”).

Finally, a few participants reported perceived challenges related to the timing FUED visit the ED. Answers reflected the idea that FUED visit the ER “at the wrong time,” “outside hours” when ER teams operate with a reduced workforce (eg, “time to dedicate with reduced workforce in the ER for instance at night”).

#### Frequency rank-order differences between professions

Perceived challenges related to FUED behavior stood in the first position across all professions and management positions. Next, the second most encountered subcategory was FUED healthcare complexity for nurses, physicians and ED staff with management positions, whereas it included FUED mental health problems for ED staff without management positions. Finally, the third position included FUED mental health problems for physicians, FUED recurrent use in nurses and ED staff with management positions and FUED chronic health problems among ED staff without management positions.

### ED Inappropriateness and Inefficiency to Address FUED Issues

Challenges related to perceived ED healthcare inefficiency were the most common answers within this category. Participants frequently reported a lack of means to properly address FUED needs in the ER. One participant evoked for instance that “ER has no means to address their [FUED’] specific social demands.” Other participants mentioned the lack of efficient treatment to address FUED issues (eg, “depending on motives, exhausted treatment options” “medically, no efficient treatment”). As a result, participants commonly pointed to the “healthcare inefficiency” to address FUED issues or to the low quality of healthcare provided (eg, “feeling of poor healthcare”). Reported reasons explaining ED inefficiency to address FUED issues commonly included shift work of ER teams (eg, “changing staff [who] does not know the patient”) resulting in “lost information” and “lack of overview.”

Similarly, the second most commonly cited subcategory included challenges related to the perception that ED is not tailored to FUED needs. Answers within this subcategory commonly reflected the idea that FUED demands are unsuitable for the ED setting (eg, “ED is most often not the right place for these patients”) most often because they are not urgent, acute (eg, “mostly chronic problems requiring specific investigation, yet noting acute”) or that they are beyond the scope of ED mission (“the patient needs to talk”).

Repetition of clinical investigations and ED healthcare futility made up the 2 next most frequently reported subcategories. Participants commonly pointed to the repetition of clinical investigations (eg, “sometimes a complete diagnostic procedure repeated every week”), which was characterized as “exaggerated,” “invasive,” inappropriate,” or “expensive.” Furthermore, repetitive investigations and ED healthcare in FUED more broadly were frequently perceived as useless. Some participants reported a waste of ED resources (eg, “waste of time,” “useless medical investigation multiplicity”) or ED occupation for unnecessary reasons (eg, “often unnecessary occupation of emergencies”).

The fifth most frequently cited challenges were related to a lack of FUED healthcare standard procedures. A few participants pointed to “the lack of FUED healthcare concept” or “guidelines” and relatedly to confusion regarding which healthcare is appropriate (eg, “[it is] unclear which measures make sense”).

Finally, the least often cited answers related to ED inappropriateness and inefficiency, including perceptions of FUED dissatisfaction. A few participants noted that FUED are dissatisfied regarding healthcare they receive in the ED (eg, “patients are discontent because they feel they receive poor healthcare,” “patient requirements are not fulfilled”).

#### Frequency rank-order differences between professions

Among the top 3 answers within this category, challenges related to the perception that ED is inefficient and not tailored to answer FUED needs stood in the 2 first positions across professions and management positions. The third position included ED over-investigation for nurses, physicians, and ED staff without management positions, whereas it comprised ED healthcare futility among ED staff with management positions.

### Lack of FUED Healthcare Network

Perceived challenges within this last category pertained to the lack of healthcare and medical follow-up outside the ED. Participants commonly mentioned FUED lack having a general practitioner, and more broadly lack medical follow-up outside the ED (eg, “lack of future ambulatory follow-up,” “we are the only one addressing their demands”). Consequently, participants noted that ED typically fulfills the absence of medical healthcare outside the ED. Finally, reported reasons explaining the lack of medical healthcare and follow-up outside the ED included the perception of poor—or a lack of—collaboration between FUED with other ambulatory medical institutions, including general practitioners (eg, “poor collaboration with alternative structures more tailored to address the patient’s problem,” “chronic case refusing a follow-up with a general practitioner”).

## Discussion

This study aimed to explore nurses and physicians’ perceptions of challenges related to FUED care in the ED of public hospitals in Switzerland. Findings revealed a wide range of challenges related to FUED care including negative consequences in the ED, FUEDs’ characteristics leading to healthcare complexity, challenges related to ED inappropriateness and inefficiency to address FUEDs needs, and lack of FUEDs’ healthcare network.

### ED Physicians and Nurses Lack Means to Properly Address FUED Needs

Consistent with past qualitative findings yielded in nurses,^19^ physicians, and nurses perceived ED as not currently set up to properly or efficiently address FUED needs. Interestingly, main findings across categories were directly or indirectly related to this challenge. First, in line with perceptions endorsed by FUED,^8,9^ they were considered as consuming lots of resources often unavailable in ED, leading to disruption of ED functioning and potentially non-satisfactory healthcare provision to FUED. Second, in line with previous findings,^8,19^ FUED demands were considered as inappropriate and often beyond the scope of ED missions, placing ED staff in the impossible position to be able to properly address them or at risk of missing a serious diagnosis. These perceptions stand in contrast with previous findings which showed that FUED consider their demands as urgent; in fact, previous research documented that FUED’ visits are mostly appropriate in light of their healthcare needs.^3,10,25^ It may be that ED staff perceive FUED demands as inappropriate because they have no means to address them efficiently.

Consistent with past literature,^26^ physicians and nurses evoked psychosocial and chronic health problems often encountered by FUED as difficult to address in ED setting. Reported reasons explaining ED inefficiency to properly address FUED demands included ED teams working in shifts resulting in a lack of overview and follow-up, lack of healthcare outside the ED and lack of standard procedures. These findings are important to consider as it may lead to both patients’ dissatisfaction regarding the care they receive and ED staff discouragement and fatigue.

### FUED is a Larger Problem Affecting Both Patients and ED Physicians and Nurses

Past qualitative research documented that ED experiences are often negative for FUED.^27^ Our findings suggest that FUED is a larger problem also affecting healthcare staff. Consistent with past findings in nurses,^19^ common reported negative consequences related to FUED care included feelings of fatigue, demotivation, discouragement and helplessness. Facing repeated visits without the possibility of properly addressing patients’ demands is likely to impact ED staffs’ sense of accomplishment and may increase risks of burnout. Burnout is common in ED physicians and nurses,^28^ and is related to negative outcomes (eg, dissatisfaction, physical and mental illness)^29^ and potentially to a decrease in empathy.^30^ This risk may be particularly important with patients considered as difficult or for whom no suitable answer exists.

### ED Staff Need Support to Address FUED Complex Needs

Taken together, findings suggest that ED staff need support to help them address FUEDs’ complex needs. Regardless of professional and management positions, some challenges were consistently endorsed, such as ED resources used by FUED, staff helplessness and fatigue, challenges related to FUED behaviors and the perception that ED is not tailored to address FUED issues and cannot address them efficiently.

One way to help address these challenges may be implementing CM. CM aims to ensure care coordination and improve patients’ healthcare empowerment, perceived self-efficacy and health literacy.^11^ CM is also likely to benefit ED staff as it facilitates referral of FUED to longitudinal care, thereby decreasing their feelings of inefficiency, helplessness, and loneliness. Finally, CM may also help address challenges related to mental health issues, an issue raised frequently among nurses and physicians without management positions. Furthermore, given the effectiveness of CM in reducing FUED visits,^14^ it may also help address challenges related to FUED use of resources over time and challenges related to FUED recurrent use, both of which were commonly endorsed by nurses and ED staff with management positions.

### Insight into How to Introduce CM Implementation to ED Staff

Descriptive findings indicated that among the participants who reported at least 1 challenge related to FUED, about 10% had previously reported they perceived FUED as a non-important problem, about 56% a rather-important problem, and about 34% an important or extremely important problem. This may indicate that some ED staff experience challenges related to FUED yet consider the situation as manageable. Similarly, preliminary findings from the ongoing parent research project^15^ indicate that about one third of the invited sites did not accept the invitation to participate and implement CM, most often because of a lack of resources or because it was not perceived as a priority. Outlining shared and specific challenges related to FUED when first introducing CM to ED staff may build a shared understanding and increase motivation to implement.

### Limitations

We used a single questionnaire item from an online survey to conduct our qualitative analysis and the resulting data were therefore limited. However, no new code emerged from the data in the coding process, indicating we reached inductive thematic saturation.^31^ Second, the questionnaire did not measure age and years of experience, which limit our ability to ensure that the sample was representative. However, respondents documented their professions and the proportions across professions in the complete sample were balanced. In addition, head physicians were the first recipients of the survey; they were asked to forward the questionnaires to ED nurses and physicians. We are unaware of the total number of staff invited to complete the survey. Although the response rate from the EDs invited to participate was acceptable (70.7%), it is possible that this process was not systematically done at the staff level. Indeed, the proportions of professions were unbalanced across linguistic regions (French speaking part sub-sample: 29 nurses [31.9%], 20 head nurses [21.9%], 27 head physicians [29.7%], and 15 residents [16.5%]; German speaking part: 19 nurses [17.3%], 20 head nurses [18.2%], 67 head physicians [61.8%], and 3 residents [2.7%]; Italian speaking part and 5 head nurses [62.5%], 2 head physicians [25%], and 3 residents [12.5%]). These differences in the proportions of professions across linguistic regions prevented us from drawing comparisons across linguistic regions. Furthermore, participation numbers varied between 1 to 19 surveys per site. That being said, the number of responses fit approximately with the number of residents in each linguistic region, although the number of hospitals contacted in the French part was slightly overrepresented. In addition, the proportions across professions in the complete sample were fairly balanced, which increases our confidence to draw comparisons between nurses and physicians. Finally, the scale of the assessment of the importance of FUED challenge was imbalanced (ie, non-important, rather important problem, important, extremely important). If giving the choice, we cannot exclude that some participants would have answered a “rather unimportant problem,” which might have impacted the answering behaviors.

## Conclusion

This study contributes to the FUED literature by describing the challenges experienced by ED nurses and physicians related to the management of FUED. Whereas past research documented negative ED experiences in FUED, findings indicate that it may represent a larger problem affecting healthcare providers as well. Implementing CM tailored to FUED might support ED staff and contribute to addressing FUEDs’ complex needs. Future research aimed at implementing CM is warranted. Considering shared and specific challenges related to FUED across professions and management position may be important when first introducing CM implementation to ED staff.

## References

[1] Pines JM Asplin BR Kaji AH , et al. Frequent users of emergency department services: gaps in knowledge and a proposed research agenda. Acad Emerg Med. 2011;18(6):e64-e69. 10.1111/j.1553-2712.2011.01086.x 21676051

[2] Malebranche M Grazioli VS Kasztura M Hudon C Bodenmann P. Case management for frequent emergency department users: no longer a question of if but when, where and how. CJEM. 2021;23(1):12-14. 33683597 10.1007/s43678-020-00024-4 PMC7726608

[3] Bieler G Paroz S Faouzi M , et al. Social and medical vulnerability factors of emergency department frequent users in a universal health insurance system. Acad Emerg Med. 2012;19(1):63-68. 22221292 10.1111/j.1553-2712.2011.01246.x

[4] Byrne M Murphy AW Plunkett PK McGee HM Murray A Bury G. Frequent attenders to an emergency department: a study of primary health care use, medical profile, and psychosocial characteristics. Ann Emerg Med. 2003;41(3):309-318. 12605196 10.1067/mem.2003.68

[5] Vu F Daeppen JB Hugli O , et al. Screening of mental health and substance users in frequent users of a general Swiss emergency department. BMC Emerg Med. 2015;15:27. 26452550 10.1186/s12873-015-0053-2 PMC4600290

[6] Huang JA Tsai WC Chen YC Hu WH Yang DY . Factors associated with frequent use of emergency services in a medical center. J Formos Med Assoc 2003;102(4):222-228. 12833184

[7] Huang JA Lai CS Tsai WC Weng RH Hu WH Yang DY. Determining factors of patient satisfaction for frequent users of emergency services in a medical center. J Chin Med Assoc. 2004;67(8):403-410. 15553800

[8] Olsson M Hansagi H. Repeated use of the emergency department: qualitative study of the patient’s perspective. Emerg Med J. 2001;18(6):430-434. 11696488 10.1136/emj.18.6.430 PMC1725740

[9] Wiklund-Gustin L. To intend to but not being able to: frequent attenders’ experiences of suffering and of their encounter with the health care system. J Holist Nurs. 2011;29(3):211-220. 21062950 10.1177/0898010110386957

[10] Wise-Harris D Pauly D Kahan D Tan de Bibiana J Hwang SW Stergiopoulos V. “Hospital was the only option”: experiences of frequent emergency department users in mental health. Adm Policy Ment Health 2017;44(3):405-412. 26961781 10.1007/s10488-016-0728-3

[11] Althaus F Paroz S Hugli O , et al. Effectiveness of interventions targeting frequent users of emergency departments: a systematic review. Ann Emerg Med. 2011;58(1):41-52. 21689565 10.1016/j.annemergmed.2011.03.007

[12] Hudon C Chouinard MC Lambert M Diadiou F Bouliane D Beaudin J. Key factors of case management interventions for frequent users of healthcare services: a thematic analysis review. BMJ Open. 2017;7(10):e017762. 10.1136/bmjopen-2017-017762 PMC5665285 29061623

[13] Di Mauro R Di Silvio V Bosco P Laquintana D Galazzi A . Case management programs in emergency department to reduce frequent user visits: a systematic review. Acta Biomed. 2019;90(6-S):34-40. 31292413 10.23750/abm.v90i6-S.8390 PMC6776176

[14] Soril LJ Leggett LE Lorenzetti DL Noseworthy TW Clement FM. Reducing frequent visits to the emergency department: a systematic review of interventions. PLoS One. 2015;10(4):e0123660. 10.1371/journal.pone.0123660 PMC4395429 25874866

[15] Grazioli VS Moullin JC Kasztura M , et al. Implementing a case management intervention for frequent users of the emergency department (I-CaM): an effectiveness-implementation hybrid trial study protocol. BMC Health Serv Res. 2019;19(1):28. 30634955 10.1186/s12913-018-3852-9 PMC6330435

[16] Chastonay OJ Lemoine M Grazioli VS , et al. Health care providers’ perception of the frequent emergency department user issue and of targeted case management interventions: a cross-sectional national survey in Switzerland. BMC Emerg Med. 2021;21(1):4. 33413163 10.1186/s12873-020-00397-w PMC7792123

[17] Schmidt M Stjernsward S Garmy P Janlov AC. Encounters with persons who frequently use psychiatric emergency services: healthcare professionals’ views. Int J Environ Res Public Health. 2020;17(3):1012. 10.3390/ijerph17031012 PMC7037678 32033481

[18] Schmidt M Garmy P Stjernsward S Janlov AC. Professionals’ perspective on needs of persons who frequently use psychiatric emergency services. Issues Ment Health Nurs. 2020;41(3):182-193. 31930924 10.1080/01612840.2019.1663565

[19] Malone RE. Almost ‘like family’: emergency nurses and ‘frequent flyers’. J Emerg Nurs. 1996;22(3):176-183. 8949217 10.1016/s0099-1767(96)80102-4

[20] Poremski D Kunjithapatham G Koh D Lim XY Alexander M Lee C. Lost keys: understanding service providers’ impressions of frequent visitors to psychiatric emergency services in Singapore. Psychiatr Serv. 2017;68(4):390-395. 27842469 10.1176/appi.ps.201600165

[21] Sandelowski M. Whatever happened to qualitative description? Res Nurs Health. 2000;23(4):334-340. 10940958 10.1002/1098-240x(200008)23:4<334::aid-nur9>3.0.co;2-g

[22] Hsieh HF Shannon SE. Three approaches to qualitative content analysis. Qual Health Res. 2005;15(9):1277-1288. 16204405 10.1177/1049732305276687

[23] Shek DTL Tang VMY Han XY . Evaluation of evaluation studies using qualitative research methods in the social work literature (1990-2003): evidence that constitutes a wake-up call. Res Soc Work Pract. 2005;15:180-194.

[24] Charmaz K. Construcing Ground Theroy: A Practical Guide Through Qualitative Analysis. SAGE Publications Ltd; 2006.

[25] Krieg C Hudon C Chouinard MC Dufour I. Individual predictors of frequent emergency department use: a scoping review. BMC Health Serv Res. 2016;16(1):594. 27765045 10.1186/s12913-016-1852-1 PMC5072329

[26] Bodenmann P Baggio S Iglesias K , et al. Characterizing the vulnerability of frequent emergency department users by applying a conceptual framework: a controlled, cross-sectional study. Int J Equity Health. 2015;14:146. 26645272 10.1186/s12939-015-0277-5 PMC4673736

[27] Hansagi H Olsson M Sjoberg S Tomson Y Goransson S. Frequent use of the hospital emergency department is indicative of high use of other health care services. Ann Emerg Med. 2001;37(6):561-567. 11385324 10.1067/mem.2001.111762

[28] Arora M Asha S Chinnappa J Diwan AD. Review article: burnout in emergency medicine physicians. Emerg Med Australas. 2013;25(6):491-495. 24118838 10.1111/1742-6723.12135

[29] Aiken LH Clarke SP Sloane DM Sochalski J Silber JH. Hospital nurse staffing and patient mortality, nurse burnout, and job dissatisfaction. JAMA. 2002;288:1987-1993. 12387650 10.1001/jama.288.16.1987

[30] Maslach C Jackson SE. The measurement of experienced burnout. J Occup Behav. 1981;2:99-113.

[31] Saunders B Sim J Kingstone T , et al. Saturation in qualitative research: exploring its conceptualization and operationalization. Qual Quant. 2018;52(4):1893-1907. 29937585 10.1007/s11135-017-0574-8 PMC5993836

